# Aptamer–gold nanoparticle-based lab-in-a-tube colorimetric detection of malathion: evaluation with classical and digital readout techniques

**DOI:** 10.55730/1300-0527.3902

**Published:** 2026-06-27

**Authors:** Emine GÜLER ÇELİK

**Affiliations:** Department of Bioengineering, Faculty of Engineering, Ege University, İzmir, Turkiye

**Keywords:** Colorimetric assays, point-of-care diagnostics, gold nanoparticle, aptamer, pesticide detection, smartphone sensing

## Abstract

Colorimetric biosensing devices represent an important class of point-of-care (POC) diagnostics due to their ability to provide visible test outputs, their ease of integration with smartphone-based sensing, and their portability for field testing. In the context of agrifood safety, there is a growing need for portable, robust, and cost-effective detection devices. Pesticide residues can be present in a wide range of environmental and food samples, from water resources to fruits and vegetables, resulting in an increasing global demand for on-site testing. Colorimetric assays, when combined with smartphone-based sensing, can effectively address these needs. In this work, the aggregation-induced color change response of aptamer–gold nanoparticle conjugates was exploited to design a lab-in-a-tube colorimetric assay for the detection of malathion. The performance of the assay was evaluated using both a conventional spectrophotometric method as a laboratory-based reference and a smartphone-based readout supported by color intensity analysis as a portable detection approach. Color responses were quantified using both a mobile application and computer software across different color channels. The smartphone-based readout yielded a limit of detection (LOD) of 5.14 mg/mL and a linear detection range of 0.02–100 mg/mL with a correlation coefficient (R^2^) of 0.9981 for the mobile application, while the computer software-based analysis exhibited an LOD of 0.88 mg/mL, a linear range of 0.02–50 mg/mL, and an R^2^ of 0.9789. In comparison, the spectrophotometric method showed an LOD of 1.67 mg/mL, a linear range of 0.02–100 mg/mL, and an R^2^ of 0.9948, confirming the reliability of the smartphone-based approaches. The results demonstrate that the colorimetric assay developed can be successfully integrated with smartphones for field-testing applications, while providing a competitive LOD comparable to laboratory-based methods.

## 1. Introduction

Colorimetric biosensing tools offer solutions for diverse applications including environmental, medical, and food analysis due to their cost-effectiveness, miniaturization and portability, ease of results interpretation, and suitability for naked-eye detection, exhibiting great potential in minimal detection technology. These properties enable intensive use of colorimetric biosensors for the development of point of care (POC) diagnostics, overcoming the limitations of complex procedures and specialized operation-based expensive instrumental setups [1]. Small metallic nanoparticles (e.g., gold nanoparticles (AuNPs) and silver nanoparticles (AgNPs)) exhibit distinct optical properties based on the localized surface plasmon resonance (LSPR) phenomenon [2]. AuNPs [3], AgNPs [4], quantum dots (QDs) [5], and polymeric nanomaterials [6] have been the most commonly investigated nanomaterials in colorimetric bioassays. Among these nanomaterials, AuNPs, which exhibit a strong LSPR absorption band in the visible region, have been widely applied in colorimetric sensing for both qualitative (naked-eye detection) and quantitative (smartphone-integrated or hand-held device-based detection) formats, as well as in commercialized products. The main advantages behind the widespread popularity of AuNPs are their ease of synthesis, versatile surface functionalization capabilities, size-dependent optical properties, and the tunability of their size and shape characteristics [7–9]. Numerous studies have demonstrated the use of AuNPs as colorimetric probes in various analytical platforms, including lateral flow assays (LFAs) [10], lab-in-a-tube assays [11], *μ*-well assays [12], paper analytical devices [13], and microfluidic systems [14]. In addition to their excellent characteristics as labeling probes, AuNPs can also act as efficient signal reporters, owing to their aggregation-induced color transition from red to purple or blue. This distinct and quantifiable shift in the UV–vis absorption spectrum enables straightforward visual detection and facilitates integration into portable and on-site diagnostic platforms [15,16]. Aptamer-conjugated AuNPs, in particular, represent an ideal class of components for POC analytical tools, as they couple the high target specificity of aptamers with the unique colorimetric responsiveness of AuNPs, rendering them particularly suitable for next-generation biosensing and diagnostic applications [17,18].

Aptamers are single-stranded DNA or RNA-based molecular recognition elements that exhibit high affinity and specificity to their targets. While aptamers are valued for their modifiability, remarkable stability, and highly programmable structures [19], they also exhibit essential advantages that make them ideal candidates for next-generation biosensor technologies: 1) Their binding-induced conformational changes make aptamers greatly functional for implementation in wearable and implantable POC devices designed for continuous monitoring. 2) The ability to design highly specific aptamers via in vitro selection for toxic, nonimmunogenic, or small molecule targets overcomes many of the inherent limitations associated with traditional biorecognition elements such as antibodies. 3) Recent advancements in the engineering of polyvalent aptamers and slow off-rate aptamers have significantly enhanced their performance and reliability in complex biological matrices [20]. A number of aptamer-based biosensors have been developed by applying various analytical techniques including electrochemical, optical, and lateral flow assay methods. Electrochemical aptasensors have successfully utilized potentiometry, differential pulse voltammetry, and cyclic voltammetry, while optical aptasensors are primarily based on fluorescence quenching and colorimetric detection approaches [21]. Although nucleic acid aptamer-based detection platforms offer significant advantages in terms of sensitivity, simplicity, and low cost, their commercialization remains limited due to performance variability across different detection matrices, which can affect the conformation, stability, and specificity of aptamers [22]. To improve practical environmental applicability and enhance their suitability for POC settings, recent studies have focused on integrating nucleic acid-based sensors with next-generation technologies such as recombinase polymerase amplification [23] and CRISPR/Cas12a-based systems [24]. In addition to performance enhancement strategies for reliable POC testing, smartphone integration has recently been studied for both electrochemical [25] and optical [26] aptasensors to improve user-friendliness and on-site applicability.

Agrifood safety analysis represents a high-potential application area for colorimetric biosensing tools, driven by the global demand for portable, rapid, user-friendly, and sustainable sensor systems [27]. Among these, pesticide residues are critical targets due to their adverse effects on ecosystems and the significant risks they pose to human health and the environment as a result of overuse and misuse [28]. In the present study, malathion was chosen as model pesticide for the design of an aptamer-based colorimetric assay. Given the well-established neurotoxic effects of malathion and its association with long-term hepatic damage, the detection of malathion residues is considered a high priority in the current literature [29].

In the present study, a solution-based colorimetric assay was developed for the detection of malathion, and its performance was evaluated using different readout techniques to quantify the color change. Specifically, the assay platform was assessed using a spectrophotometer as a conventional readout system and a smartphone camera as a digital readout system. Additionally, for smartphone camera-based quantification, two color intensity measurement platforms were employed: a computer-based platform and a mobile application-based platform. To generate both visible and quantifiable colorimetric signals, the aggregation-induced color change properties of AuNPs were exploited. Specific biorecognition was achieved through the conjugation of aptamers onto AuNPs. According to the literature, the assay system can be described as a “lab-in-a-tube” assay, a concept previously proposed by Merkoçi’s group, owing to its solution-based colorimetric nature [11]. The assay system exhibited reliable performance in malathion standard solutions across different readout methods and in malathion-spiked tap water samples, enabling the evaluation of matrix effects.

## 2. Materials and methods

### 2.1. Materials

Gold nanoparticle solution (40 nm) was purchased from Cytodiagnostics Inc. (Burlington, ON, Canada). The malathion aptamer sequence (5′-SH-ATCCGTCACACCTGCTCTTATACACAATTG-TTTTTCTCTTAACTTCTTGACTGCTGGTGTTGGCTCCCGTAT-3′) was chosen according to a previous study [16] and was obtained from Metabion International AG (Planegg, Germany). Malathion standard was purchased from Sigma Aldrich. All reagents and chemicals were analytical grade.

### 2.2. Instrumentation

A BioTek Epoch 2 model microplate spectrophotometer (Agilent Technologies Inc., Santa Clara, CA, USA) was used for UV–vis absorbance measurements. A Malvern NanoZS model zeta sizer system (Malvern Panalytical Ltd., Malvern, Worcestershire, UK) was used for both particle size and zeta potential measurements.

### 2.3. Aptamer–gold nanoparticle conjugation

Prior to the conjugation reaction, AuNPs (OD = 1) were preconcentrated by centrifugation to enhance naked-eye visibility during assay preparation. AuNPs dispersed in sodium phosphate buffer (10 mM, pH 7.0) containing Tween 20 were incubated on a shaker at room temperature and 1000 rpm for 40 min, followed by centrifugation at 4 °C and 4500 rpm for 20 min. Subsequently, 25 μL of malathion aptamer (100 μM) was added to the AuNP suspension, and the reaction mixture was incubated for 3 h at 1000 rpm. Finally, the mixture was centrifuged at 4 °C and 4500 rpm for 20 min to remove unconjugated aptamers [30]. The resulting Apt-AuNP conjugates were stored at 4 °C for further use.

### 2.4. Characterization of Apt-AuNP conjugates

The successful modification of AuNPs with aptamers was evaluated through UV–vis absorbance analysis, particle size measurements, and zeta potential characterization.

### 2.5. Lab-in-a-tube assay principle

Citrate-stabilized AuNPs tend to aggregate upon interaction with positively charged ions (e.g., Na^+^, Mg^2+^, and Ca^2+^) due to their negatively charged surfaces. In contrast, surface modification with biomolecules such as aptamers enhances the colloidal stability of AuNPs against aggregation induced by positively charged ions. Upon aggregation, the color of AuNPs shifts from red/pink to purple/blue, a change that is readily visible to the naked eye. Based on these principles, AuNP aggregation-induced assays are considered practical and feasible approaches according to the literature [31]. Briefly, in the assay, the water sample to be analyzed is added to a microtube containing a malathion aptamer-modified AuNP solution, followed by the addition of NaCl solution. The resulting color change is recorded using a smartphone camera. In the presence of malathion, the red/pink color of the solution shifts to purple/blue. The intensity of this color change is used for the quantitative determination of malathion in the water sample (Scheme a).

As illustrated in Scheme b, when a sample containing malathion is added to the Apt-AuNP solution, a specific aptamer–malathion interaction occurs. Owing to the strong affinity of this interaction, the aptamers lose their ability to effectively protect the AuNP surface from salt-induced aggregation. Consequently, in the presence of NaCl, the AuNPs aggregate and precipitate. In contrast, when the applied sample does not contain malathion, the aptamers remain adsorbed on the AuNP surface and maintain nanoparticle stability under NaCl conditions, preventing aggregation. The color change is proportional to the amount of malathion in the sample; as the malathion concentration increases, AuNP aggregation increases accordingly, resulting in a more pronounced color change from red to purple.

### 2.6. Assay optimization

In this step, the NaCl concentration and treatment time were optimized to obtain both visually observable and quantifiable colorimetric responses. For this purpose, 10 μL of NaCl solutions at different concentrations (0, 15, 30, 60, 120, and 150 mM) [31] was added to both AuNPs and Apt-AuNP conjugates (100 μL). Subsequently, color intensity changes were evaluated at 5, 15, 20, 30, and 50 min using image capture and UV–vis spectrophotometric measurements at 530 nm.

### 2.7. Assay performance

To construct calibration curves, malathion solutions at concentrations of 0.01, 0.02, 0.05, 0.1, 10, 20, 50, and 100 mg/mL were added to wells containing 100 μL of Apt-AuNP conjugates, and the well plate was incubated at 300 rpm for 30 min. Subsequently, 0.6 M NaCl was added to each well, and the resulting color changes were measured using both a smartphone camera and a UV–vis spectrophotometer. Color analysis of the captured images was performed using a mobile application (Color Analysis Version 4, Google Play) and computer-based software (ImageJ 1.54g). All images were captured under identical ambient lighting conditions using the same smartphone camera at a fixed distance and angle throughout the experiments. Color analysis was performed using both the Color Analysis application and ImageJ software from three independent regions of each image. For the Color Analysis application-based analysis, magenta intensity values were recorded from three different regions of each image and averaged. For the ImageJ-based analysis, the mean blue intensity values were obtained from the RGB histogram of the selected regions. These values were used as quantitative colorimetric readout parameters for calibration and comparison. To evaluate the practical applicability of the assay, malathion-spiked tap water samples were also analyzed.

## 3. Results

### 3.1. Characterization studies

**Figure 1:**
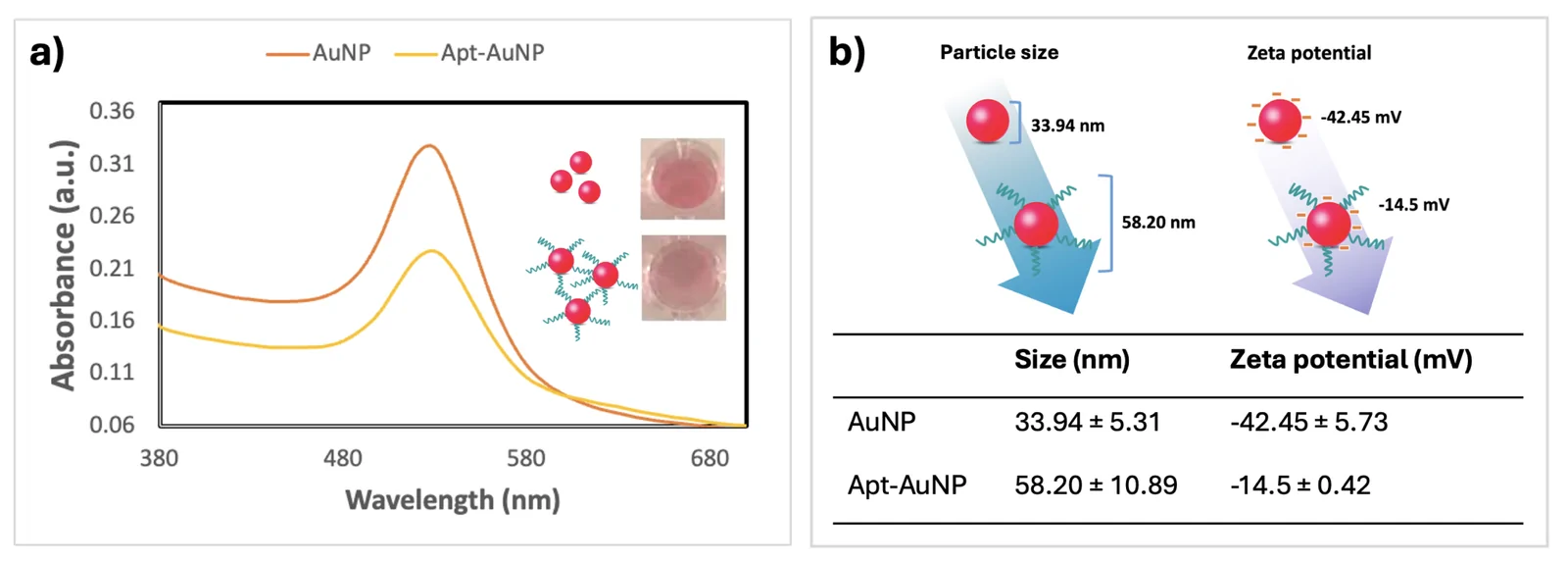
a) Spectrophotometric measurements and b) the results of particle size and zeta potential of AuNPs and Apt-AuNP conjugates.

To confirm aptamer conjugation on the surface of AuNPs, spectrophotometric measurements were performed. As shown in Figure 1a, citrate-stabilized AuNPs exhibited a characteristic surface plasmon resonance peak at 530 nm, consistent with AuNPs of approximately 40 nm in diameter [32]. Following aptamer conjugation, a decrease in peak intensity accompanied by a slight red shift was observed, indicating successful surface modification.

Figure 1b presents the particle size and zeta potential values of AuNPs and aptamer-modified AuNPs. Following conjugation, the particle size increased from 33.94 nm to 58.20 nm, while the zeta potential shifted from −42.45 mV to −14.5 mV, confirming successful surface modification. The negative surface charge of bare AuNPs originates from citrate capping, whereas surface functionalization with aptamers reduces the net negative charge. The observed increase in particle size is attributed to the attachment of oligonucleotide aptamers onto the AuNPs’ surface [33].

### 3.2. Optimization results

**Figure 2:**
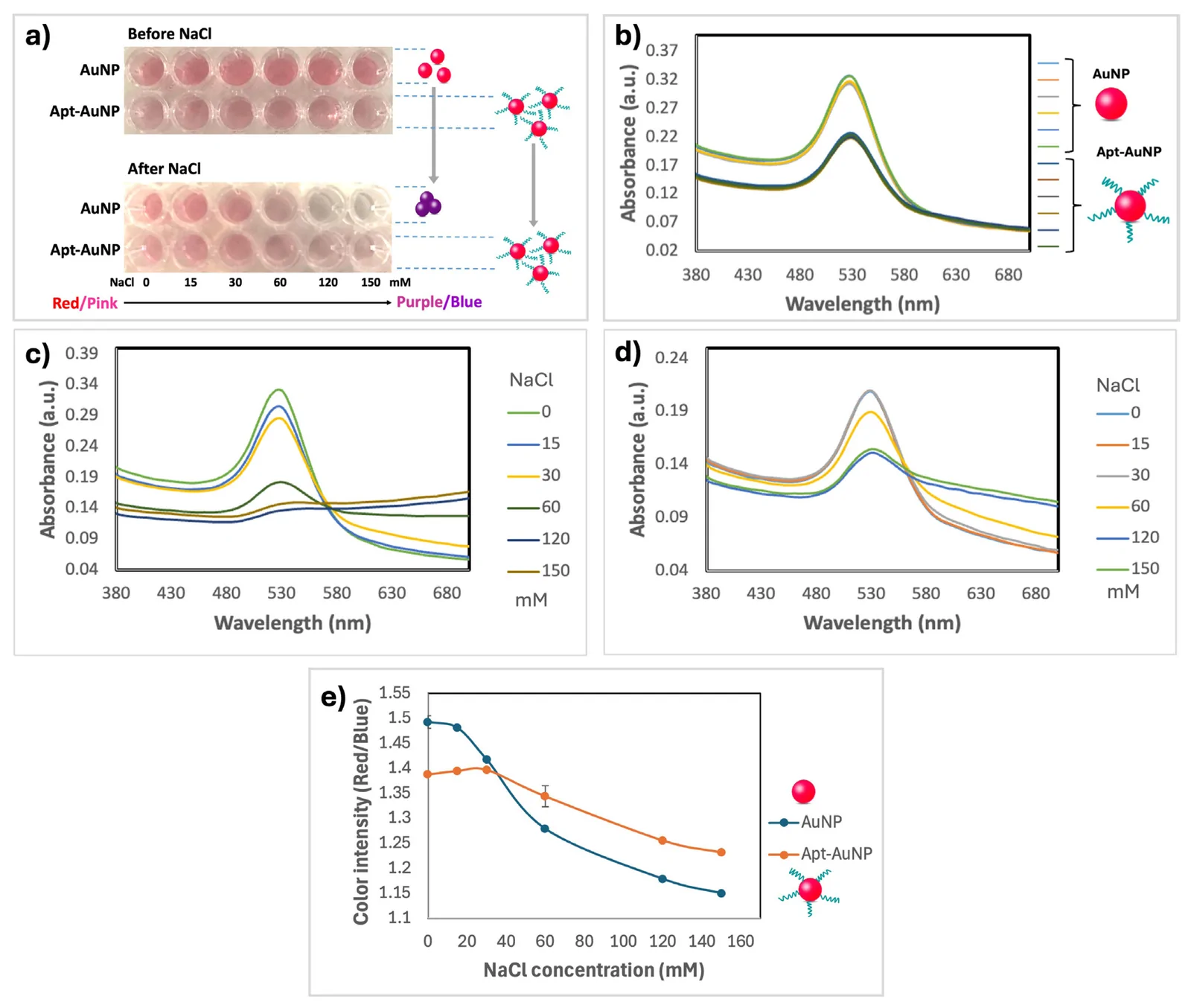
a) Photographs of wells containing AuNPs and aptamer-modified AuNPs (Apt-AuNPs) before and after NaCl treatment. b) UV–vis absorbance spectra of wells containing AuNPs and Apt-AuNPs prior to NaCl addition. UV–vis absorbance spectra of NaCl-treated c) AuNPs and d) Apt-AuNPs. e) Red to blue color intensity ratio of wells after NaCl addition.

First, the NaCl concentration was optimized, as the assay principle relies on aggregation-induced color changes. For this purpose, NaCl solutions at concentrations of 0, 15, 30, 60, 120, and 150 mM were added to wells containing either AuNPs or Apt-AuNPs. As shown in Figure 2a, the color of bare AuNPs gradually changed from red to purple with increasing NaCl concentration, indicating salt-induced aggregation. In contrast, Apt-AuNP conjugates were only slightly affected, as expected, due to surface functionalization with aptamers.

In addition to the visible color changes, the absorbance spectra of both AuNPs and Apt-AuNPs before and after NaCl treatment were recorded using a UV–vis spectrophotometer. As presented in Figure 2b, prior to NaCl addition, AuNPs and Apt-AuNPs each exhibited characteristic absorbance peaks around 530 nm, with consistent intensities within their respective wells. However, increasing NaCl concentrations caused a significant decrease and red shift in the absorbance peaks of bare AuNPs, indicating aggregation (Figure 2c). In contrast, the absorbance intensities of Apt-AuNPs were only slightly affected at higher NaCl concentrations (60, 120, and 150 mM) (Figure 2d). Figure 2e shows the red-to-blue color intensity ratio of the wells, calculated using ImageJ, and represents a quantitative metric for the aggregation-induced red-to-blue/purple color change. Based on these results, a NaCl concentration of 60 mM and 15 min treatment time was selected for further assay development, as this concentration induced aggregation of bare AuNPs while maintaining the stability of Apt-AuNP conjugates.

### 3.3. Lab-in-a-tube assay: Performance studies

To assess the adaptability of the colorimetric assay developed to different readout systems, which enables flexibility across various field-application scenarios, calibration curves were generated using conventional spectrophotometry and smartphone-based analyses performed via mobile applications and computer software.

#### 3.3.1. Conventional spectrophotometric analysis

**Figure 3:**
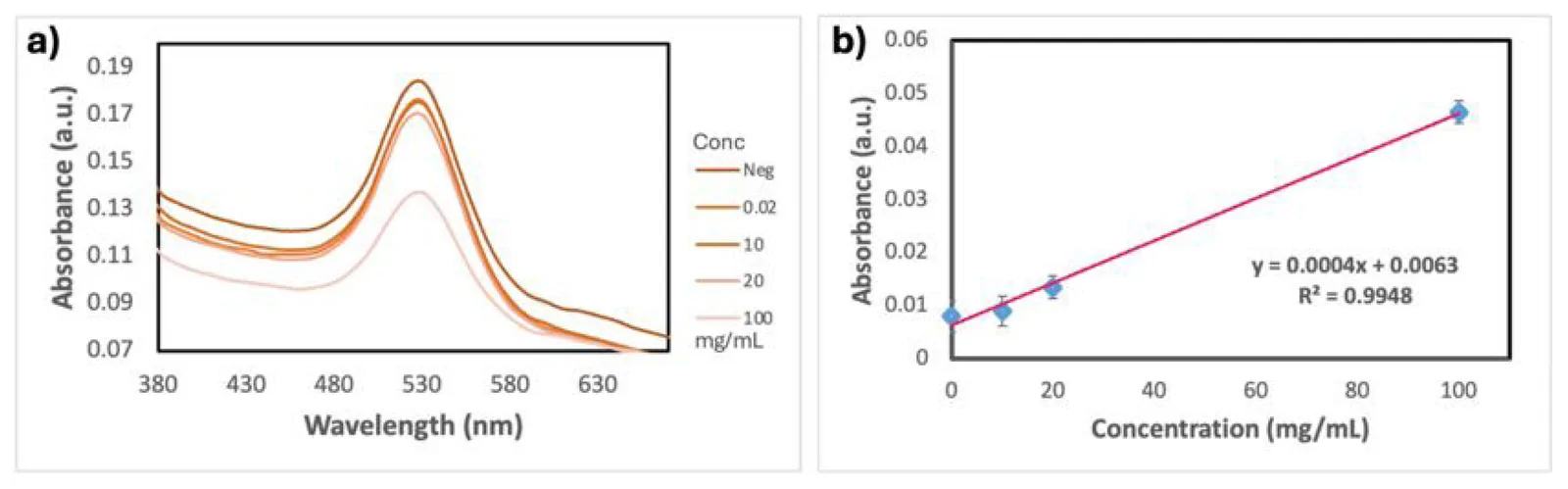
a) UV–vis absorbance spectra of test solutions treated with increasing concentrations of malathion. b) Calibration curve for malathion detection obtained from spectrophotometric measurements. Data are presented as mean ± standard deviation (SD) obtained from three independent measurements (n = 3).

In the assay designed, increasing malathion concentrations resulted in a gradual decrease in absorbance intensity (Figure 3a). Based on the absorbance spectra, a calibration curve was constructed over the concentration range of 0.02–100 mg/mL, exhibiting good linearity (Figure 3b).

#### 3.3.2. Smartphone-based digital readout

Smartphone-based detection technologies have accelerated the development of portable diagnostic devices. Following image acquisition, color intensity analysis can be performed using either mobile applications or computer-based software. The colorimetric responses obtained from the assay were evaluated by analyzing changes in the red–green–blue (RGB) color channels. As the assay generates signals through the formation of a purple/blue color with increasing malathion concentration, accompanied by a decrease in red/pink coloration, the blue and magenta color intensities were monitored during the image analysis process. Initially, different color channels were comparatively evaluated to determine the most suitable colorimetric response for each image analysis approach. For the ImageJ-based analysis, red and blue channel intensities obtained from RGB histograms were investigated, while magenta, red, pink, and blue intensity parameters were evaluated using the Color Analysis mobile application. Among the color channels evaluated, magenta was identified as the optimal target signal for mobile application-based analysis (Color Analysis), whereas blue was found to be the most suitable channel for computer-based analysis using ImageJ, as these channels exhibited more distinct concentration-dependent responses compared to the other parameters evaluated.

**Figure 4:**
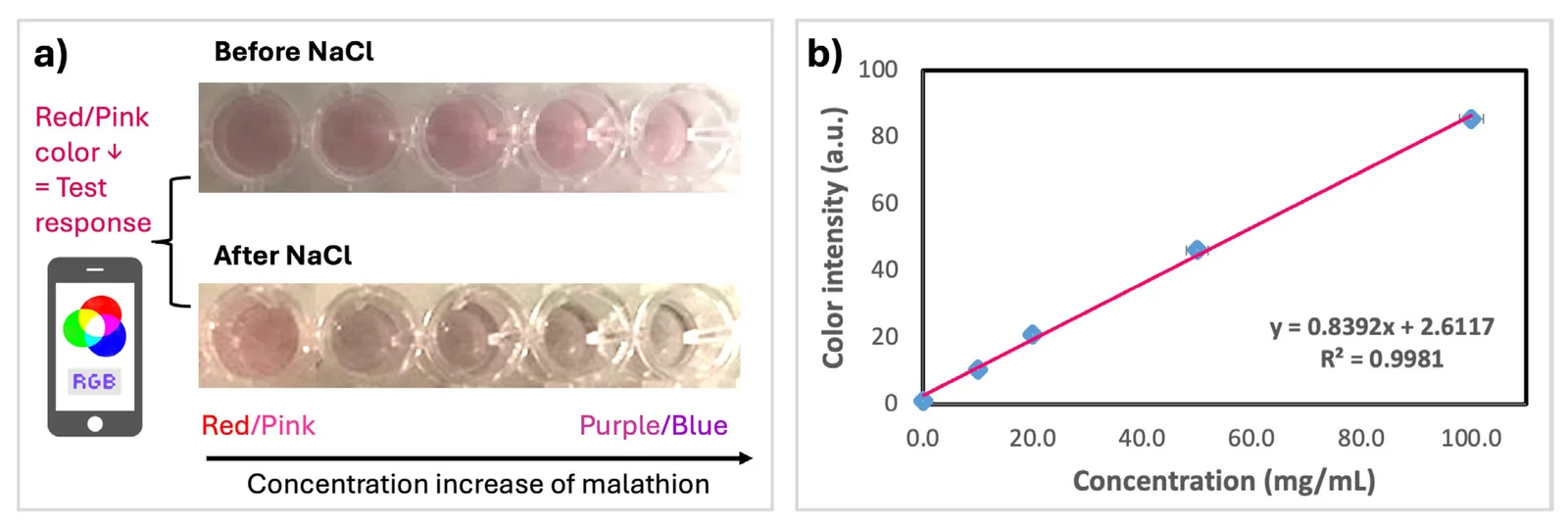
a) Color changes of test solutions treated with increasing concentrations of malathion before and after NaCl application. b) Calibration curve for malathion constructed using the decrease in magenta color intensity as the analytical response obtained from mobile application-based image analysis. Color intensity values were obtained from three different regions of each photograph and are presented as mean ± SD (n = 3).

For the first approach, magenta color intensities of test solutions treated with increasing concentrations of malathion were measured before and after NaCl addition using a mobile application (Figure 4a). Subsequently, the differences in color intensity were used to construct a concentration-dependent calibration curve. As presented in Figure 4b, a linear calibration curve was obtained with a strong regression coefficient (R^2^ = 0.9981).

**Figure 5:**
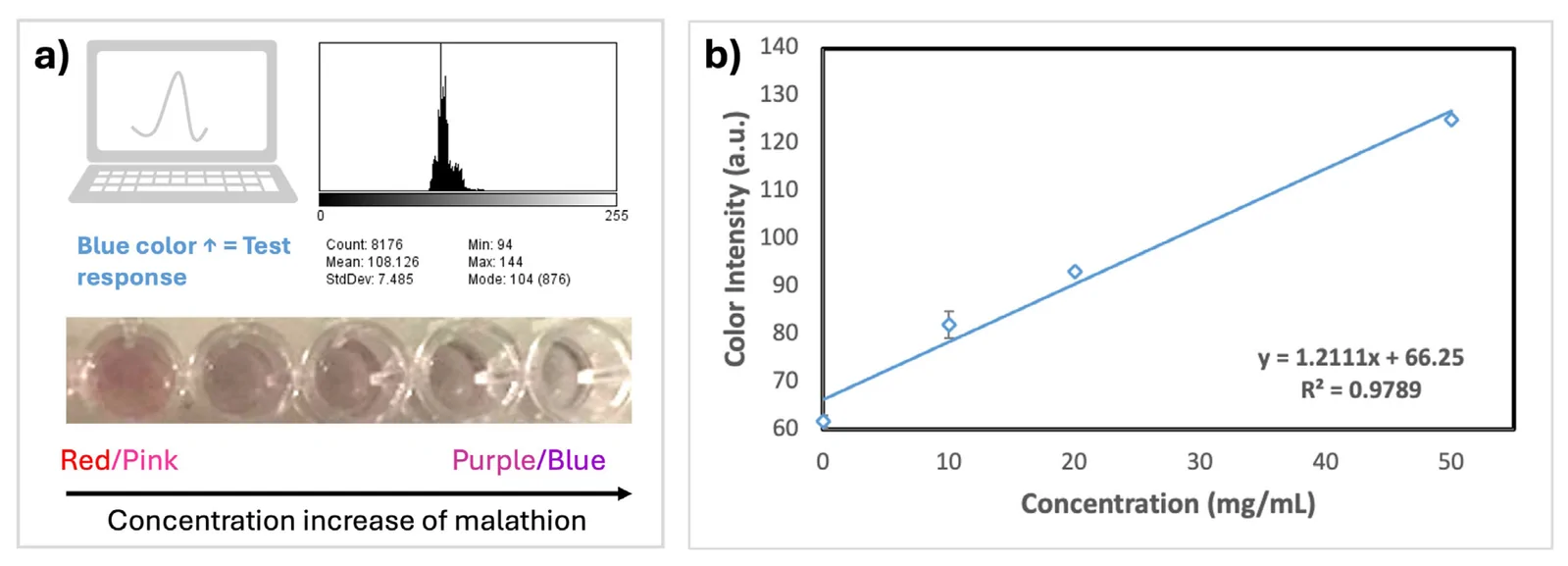
a) Photographic images showing the color change from red/pink to purple/blue with increasing malathion concentration. b) Calibration curve for malathion constructed using the increase in blue color intensity as the analytical response obtained from ImageJ-based image analysis. Color intensity values were obtained from three different regions of each image and are presented as mean ± SD (n = 3).

In the second approach, the increase in blue color intensity was quantified using ImageJ, as the assay produces enhanced blue coloration in parallel with increasing malathion concentrations (Figure 5a). A calibration curve was generated over the linear concentration range of 0.02–50 mg/mL (Figure 5b).

When the assay performance was evaluated according to the different readout strategies (Table 1), the spectrophotometric method provided a linear response over the concentration range of 0.02–100 mg/mL with a strong correlation coefficient (R^2^ = 0.9948). Computer software-based image analysis (ImageJ) exhibited higher sensitivity; however, it showed a narrower linear range (0.02–50 mg/mL) and a slightly lower correlation coefficient (R^2^ = 0.9789). In contrast, mobile application-based quantification demonstrated excellent linearity (R^2^ = 0.9981) across a wide concentration range of 0.02–100 mg/mL.

In terms of the limits of detection (LOD), computer software-based image analysis yielded the lowest LOD value (0.88 mg/mL), indicating superior analytical sensitivity. The spectrophotometric readout, with an LOD of 1.67 mg/mL, reflected its role as a reliable reference method. Although mobile application-based analysis resulted in a higher LOD (5.14 mg/mL), it offers a robust and field-deployable alternative for on-site malathion detection. Notably, the performance of the mobile application-based approach could be further improved through optimization of lighting conditions and camera sensitivity and integration of advanced image-processing or machine learning algorithms.

To test the field application potential of the assay developed, malathion-spiked tap water samples were applied, and the results were obtained using a mobile application-based readout. According to the test responses, 23.74 mg/mL and 49.28 mg/mL malathion were detected in the samples spiked with 20 mg/mL and 50 mg/mL malathion, respectively, with recovery values of 118.7% and 98.56%. These recovery experiments provided an initial indication of applicability in real samples within the scope of a proof-of-concept demonstration. However, further studies involving additional concentration levels, increased replicates, and more diverse real sample matrices are required for the development of a fully validated malathion detection kit.

Table 2 presents a systematic comparison of the current study with previously reported AuNP/aptamer-based colorimetric malathion sensing platforms. Although several studies in the literature are based on aggregation-induced color changes of AuNPs, their sensing strategies and signal generation mechanisms differ from those employed in the present work. Compared with the studies summarized in Table 2, the current study is unique in integrating smartphone-based readout approaches for colorimetric sensing, despite operating within relatively high concentration ranges. Since the primary objective of our work was not the development of a fully optimized malathion detection kit, a direct analytical performance comparison may not fully reflect the intended scope of the study. Rather, the main contribution of our work is the demonstration of smartphone-assisted color analysis concepts in an aptamer-based lab-in-a-tube sensing platform.

## 4. Discussion

The present work involves the design of an aptamer-based colorimetric lab-in-a-tube assay for the detection of malathion and the evaluation of colorimetric signals using different readout systems, including conventional spectrophotometric measurement and smartphone-based readout. The assay successfully generated visible and measurable colorimetric signals over a malathion concentration range of 0.02–100 mg/mL. Assay images captured using a mobile phone were processed using both a mobile application and computer software to quantify color signals. While the mobile application provided reliable results based on magenta color intensity, the computer software exhibited optimal signal response in the blue channel. The results demonstrate that AuNPs, when combined with aptamers as specific biorecognition elements, act as effective nanoprobes that can be integrated into smartphone-based diagnostics for in-field testing with portable devices. The present study contributes to the literature by demonstrating that smartphones can serve as alternative detection systems under laboratory-independent conditions and in resource-limited settings. However, the malathion concentration range tested reflects a proof-of-concept evaluation rather than direct applicability to real samples, as the primary aim of our study was not to present a fully optimized malathion detection platform. Therefore, further optimization of the assay sensitivity using environmentally relevant and more evenly distributed concentration levels, together with comprehensive cross-reactivity studies involving other organophosphate pesticides, is necessary for practical field applications. Additionally, further studies involving standardized imaging approaches, such as the use of a light box, calibration cards, or controlled illumination systems, are required to validate and standardize smartphone-integrated measurements against environmental lighting variations.

## Figures and Tables

**Scheme:**
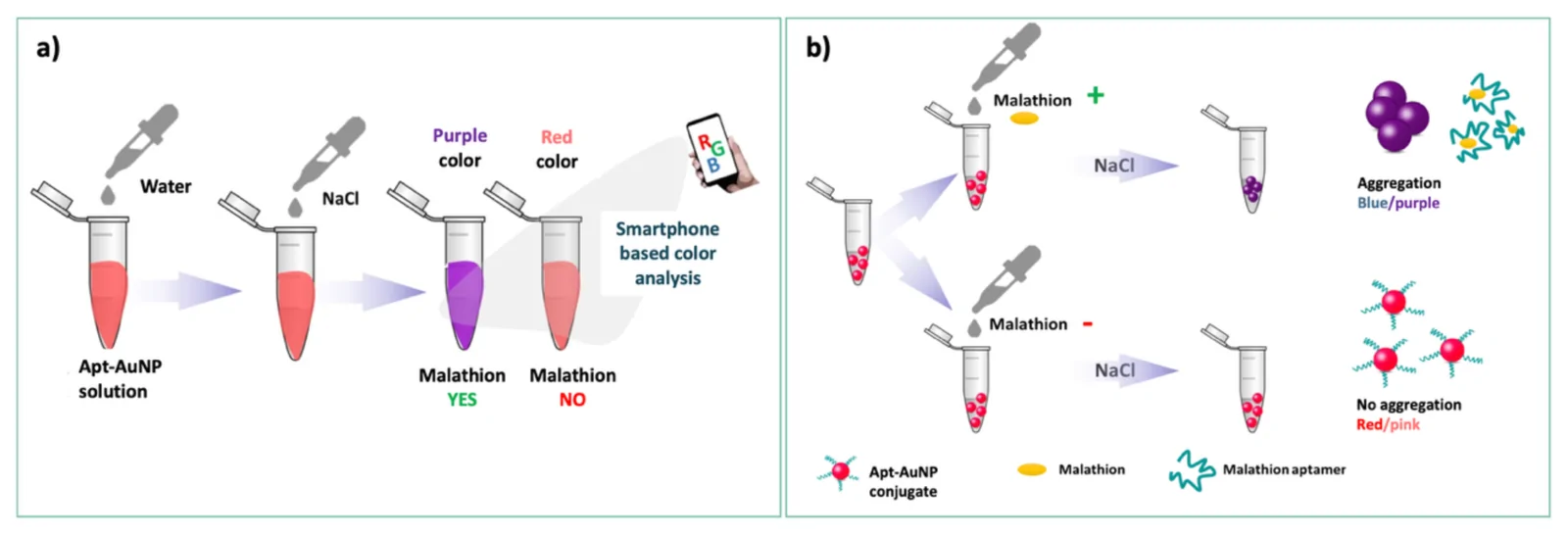
a) Basic working principle of the aptamer–gold nanoparticle-based lab-in-a-tube colorimetric assay. b) Schematic representation of the signal generation mechanism.

**Table 1:** Analytical performance comparison of the developed assay using different readout strategies. LOD values were estimated based on linear regression analysis using the equation LOD = 3.3σ/S, where σ is the standard error of the intercept and S is the slope of the calibration curve.

Readout Method	Linear Range (mg/mL)	Regression Equation	R^2^	Slope	LOD (mg/mL)
**Spectrophotometer**	0.02–100 mg/mL	y = 0.0004x + 0.0063	0.9948	0.0004	1.67
**Mobile Application (Magenta Channel)**	0.02–100 mg/mL	y = 0.8392x + 2.6117	0.9981	0.8392	5.14
**Computer Software (ImageJ, Blue channel)**	0.02–50 mg/mL	y = 1.2111x + 66.25	0.9789	1.2111	0.88

**Table 2:** Comparison of the present work with AuNP/aptamer-based malathion studies.

Platform / sensing strategy	Readout strategy	Linear range	LOD	Main practical advantage	Ref.
Aptamer–PDDA–AuNP colorimetric aptasensor	Visual / UV–Vis colorimetric	0.5–1000 pM	0.06 pM	Very sensitive	[16]
dsDNA-modified AuNP aggregation based on target-induced hairpin formation	A650/A520 UV–Vis ratio	5.0 pM–10 nM	1.0 pM	Hairpin-assisted specificity and low LOD	[34]
Aptamer/protamine/AuNP visual colorimetric assay	Naked-eye / UV–Vis / DLS	0–50 μg/L	1.48 μg/L	Mix-and-measure assay within 40 min	[35]
Fe-MOF-based aptasensor with catalytic amplification	Colorimetric + electrochemical dual-output	10–500 ng/mL	5.8 ng/mL colorimetric; 4.6 ng/mL electrochemical	Dual-signal verification improves reliability	[36]
Aptamer–AuNP lab-in-a-tube colorimetric system	Spectrophotometric, Smartpone sensing (mobile application-based, and ImageJ-based digital color analysis)	0.02–100 mg/mL	1.67 mg/mL spectrophotometer; 5.14 mg/mL Mobile Application; 0.88 mg/mL Computer Software	Comparative evaluation of classical and digital readout strategies in a simple portable format with potential suitability for smartphone-assisted POC integration in portable testing applications	This study

## Data Availability

Data supporting the findings of this study are contained within the article. Any additional raw data may be made available by the corresponding author upon reasonable request.
